# Review

**Published:** 1871-10

**Authors:** W. S. Armstrong

**Affiliations:** Late Professor of Anatomy in the Atlanta Medical College


### REVIEW.

BY W. S. ARMSTRONG, M.D.,
Late Professor of Anatomy in the Atlanta Medical College.

Reflections on the Anatomy and Physiology of Some Parts of the Eye. By R. F. Michel, M.D., Ex-President of the Medical Association of the State of Alabama, Vice-President of the American Medical Association. Montgomery, Alabama. 1871.

That the organ of vision is entirely deprived of lymphatics, no well-informed anatomist or physiologist will deny. This fact naturally leads to the inquiry as to the manner in which the secretion and nutrition of the eye is performed.

In this neat pamphlet, the author sets forth in a clear light what, one quarter of a century ago, were his published convictions of the manner in which these functions were performed. In the outset, he gives, as the result of his investigations, only so much of the anatomical structure of the eye as is necessary to clearly elucidate the subject, and from them, by a happy process of reasoning, deduces the mode, as he believes, in which these important offices are performed. After mentioning the layers of the choroid—the venous, arterial and pigmentous—he directs special attention to the manner of their termination at the anterior part of the eye. From injections into the carotid artery and jugular vein, he concludes that the internal coat of the *iris* is but a continuation of the *choroid*, and consequently vascular; that the *ciliary process* is likewise a continuation of the choroid, and vascular. Of this there seems to be but little doubt, at the present day, among physiologists. Yet there are some—and among them stand conspicuously Todd and Bowman—who still assert that the ciliary processes, or the vessels which form them are covered or accompanied by a muscular layer.

The *iris* and *ciliary processes*, then, he regards as appendages of the *choroid*. The ciliary ligament—or muscle, as he terms it—unites the anterior muscular layer of the iris to the choroid, and at the same time forms an attachment for the two latter to the sclerotic.

It will be understood, then, that the vitreous is enveloped, as it were, by a most remarkable coat, formed entirely of blood-vessels; but in no instance, in making injections into them, did the hyaloid membrane of the vitreous humor give evidence of being traversed by blood-vessels. The vascularity of the eye, then, is abundantly proved until the vitreous is reached; and he, therefore, agrees with Stellwag, who says, in speaking of the vitreous: "*It is perfectly structureless, without vessels and nerves.*"

Regarding, then, he says, the humors of the eye as completely isolated from the rest of that organ—being enveloped by their proper membranes, which, preserving the character of true serous membranes, are remarkable for their extreme tenuity and non-vascularity—the question arises: "*In what manner is secretion, nutrition and absorption effected?*"

These offices, by a most happy process of reasoning, the details of which we have not space to reproduce here, he assigns to the venous system of the choroid—more particularly to the ciliary processes, which were demonstrated to be so intimately connected with the vitreous humor. In support of his theory, he refers to the experiments of Magendie, who demonstrated, by a series of experiments, that the blood-vessels do absorb. In support of Dr. Michel's position, we think the most conclusive evidence we have of absorption by the veins, is the experiment of Panizza on the horse. He introduced hydrocyanic acid into a section of the animal's intestine entirely separated from the body, except by an artery and vein, which were necessary to carry on the circulation. So long as the vein was compressed, no effect was produced; but when the pressure was removed from the vein, there were evidences of poisoning—besides, blood taken from the vein was found to contain hydrocyanic acid.

We take pleasure in recommending this ably-written pamphlet to those interested in the subject.

